# Drug-Induced Liver Injury in COVID-19 Patients: A Systematic Review

**DOI:** 10.3389/fmed.2021.731436

**Published:** 2021-09-20

**Authors:** Fatemeh Sodeifian, Zahra Sadat Seyedalhosseini, Naghmeh Kian, Mahya Eftekhari, Shaghayegh Najari, Mehdi Mirsaeidi, Yeganeh Farsi, Mohammad Javad Nasiri

**Affiliations:** ^1^Student Research Committee, School of Medicine, Shahid Beheshti University of Medical Sciences, Tehran, Iran; ^2^School of Medicine, Shahid Beheshti University of Medical Sciences, Tehran, Iran; ^3^School of Dentistry, Shahid Beheshti University of Medical Sciences, Tehran, Iran; ^4^Division of Pulmonary and Critical Care, Department of Medicine, University of Miami Miller School of Medicine, Miami, FL, United States; ^5^Department of Pulmonary and Critical Care, Miami VA Medical Center, Miami, FL, United States; ^6^Department of Microbiology, School of Medicine, Shahid Beheshti University of Medical Sciences, Tehran, Iran

**Keywords:** COVID-19, SARS-CoV-2, drug induced liver injury (DILI), liver injury, adverse drug reaction

## Abstract

**Introduction:** The severity of COVID-19 may be correlated with the risk of liver injury development. An increasing number of studies indicate that degrees of hepatotoxicity has been associated with using some medications in the management of COVID-19 patients. However, limited studies had systematically investigated the evidence of drug-induced liver injury (DILI) in COVID-19 patients. Thus, this study aimed to examine DILI in COVID-19 patients.

**Methods:** A systematic search was carried out in PubMed/Medline, EMBASE, and Web of Science up to December 30, 2020. Search items included “SARS-CoV-2”, “Coronavirus,” COVID-19, and liver injury.

**Results:** We included 22 related articles. Among included studies, there was five case report, five case series, four randomizes control trial (RCT), seven cohort studies, and one cross-sectional study. The drugs included in this systematic review were remdesivir, favipiravir, tocilizumab, hydroxychloroquine, and lopinavir/ritonavir. Among included studies, some studies revealed a direct role of drugs, while others couldn't certainly confirm that the liver injury was due to SARS-CoV-2 itself or administration of medications. However, a significant number of studies reported that liver injury could be attributable to drug administration.

**Discussion:** Liver injury in COVID-19 patients could be caused by the virus itself or the administration of some types of drug. Intensive liver function monitoring should be considered for patients, especially patients who are treated with drugs such as remdesivir, lopinavir/ritonavir, and tocilizumab.

## Introduction

Today, Severe Acute Respiratory Syndrome Coronavirus 2 (SARS-CoV-2) infection, causing the pandemic Coronavirus Disease 2019 (COVID-19), a novel acute respiratory disease, which has affected 220 countries and territories with more than 200 million infected individuals and more than 4 million deaths, has become a serious global health concern.

With the main clinical manifestations of cough, fever, and shortness of breath (1), and the respiratory tract being the leading site of infection, the course of the disease is complex in a portion of the cases, in which it involves multi-organ including liver (2). As angiotensin-converting enzyme 2 (ACE2) is the central receptor for SARS-CocV-2 entry to the host cells (3, 4), its wide distribution in different body tissues can explain multi-organ involvement in COVID-19. Epidemiological studies (3) indicate different degrees of elevated liver chemistries with an incidence of 24.4%, particularly in liver transaminases, Aspartate transaminase (AST), and Alanine aminotransferase (ALT) in COVID-19 patient (5). COVID-19 associated liver injury, defined as any damage that occurred to the liver due to pathogenesis or treatment of COVID-19 (6), has been reported to occur in 20–46.9% of the COVID-19 patient (7, 8).

It's been shown that the severity of COVID-19 is correlated with the risk of liver injury development (8, 9). Furthermore, it's been suggested that liver injury is associated with poor outcomes of SARS infection, which is still a matter of debate (10, 11). Besides, the CT-quantified liver/spleen attenuation ratio has further proved the liver damage in COVID-19 patients, which was correlated with the severity of the disease (12). In previous studies, SARS-CoV viral particles have been identified in hepatocytes (13), and direct induction of liver injury by SARS-CoV was observed *in vitro* (14). In addition, SARS-CoV-2 is also shown to be associated with Liver tissue damage and dysfunction (15). While being mild in most cases, these liver manifestations of COVID-19 can potentially cause some adverse effects, from blood coagulation abnormalities causing severe bleeding to liver failure and even death caused by liver function deterioration (15–17). Hence, it is essential to find out the underlying mechanisms of these liver manifestations to prevent such adverse effects.

Since its initial rise in December 2019, various therapeutic compounds have been used to control the progression of pathogenesis and symptoms in the course of COVID-19. This drug armamentarium consists of several groups: (1) Antiviral drugs, including remdesivir, lopinavir/ritonavir, favipiravir, triazavirin, and umifenovir, (2) Antibiotics, including azithromycin and ceftriaxone, (3) Antimalarials, mainly hydroxychloroquine, (4) Immunomodulator agents, including tocilizumab and steroids like Dexamethasone, (5) antipyretic medications like acetaminophen, and (6) other adjunctive treatments like zinc sulfate and vitamin C, and several investigational treatments including convalescent plasma administration from COVID-19 recovered individuals and high-dose anakinra (an IL-1β inhibitor) (18). Previously some degrees of hepatotoxicity have been reported for many of these therapeutic agents as they were used for other diseases like viral infections.

An increasing number of studies indicate that degrees of hepatotoxicity have been associated with using some of these medications in the management of COVID-19 patients. Significantly, it was relieved after the cease of these agents. However, to our best knowledge, no studies had systematically investigated the evidence of drug-induced liver injury (DILI) in COVID-19 patients until today. In this study, to elucidate the association between hepatotoxicity in COVID-19 patients and the drugs used in these patients and to better identify the role of DILI as a possible mechanism of hepatotoxicity, the currently available evidence on the association of different therapeutic agents with hepatotoxicity in COVID-19 patient was systematically reviewed.

## Methods

This systematic review was conducted based on the “Preferred Reporting Items for Systematic Reviews and Meta-Analyses” (PRISMA) statement (19).

### Search Strategy

Our team performed systematic literature searches through PubMed/Medline, Web of Science, and Embase databases. We included case reports and case series for the article type published until January 2021. Search items included “SARS-CoV-2”, “Coronavirus”, “COVID-19”, and “liver injury”. We included studies written in English.

### Eligibility Criteria

We reviewed any studies reporting liver injury and liver-related adverse events caused by drug administration in COVID-19 patients including case reports, case series, case- controls, cohorts, clinical trials and observational studies. Definite adult cases of COVID-19 (mainly via a positive COVID-19 PCR) whom were hospitalized considered as the target population of our study. The studies that reported liver injury due to SARS-CoV-2 were not included in this study.

### Study Selection

To find eligible articles, we screened potentially valid papers through two steps, step one reviewing the title and the abstract of the articles, and step two, reviewing the full text of the qualified articles from the first step. In both steps, each article was screened by two reviewers independently. If any ambiguity or disagreement was met, it was discussed among authors, and a final decision was made.

### Data Extraction

We extracted the following as data: first author, publication year, type of study, the country was the study was conducted, mean age, medications, COVID-19 symptoms, patients comorbidities, inclusion and exclusion criteria, number of charged/discharged patients, the severity of COVID-19 disease, and laboratory liver function tests. In addition, we extracted demographic data of patients in the medication group but not in the placebo group. Similarly, for this step, each article data sheet was completed and reviewed by two authors independently, and disagreements and technical uncertainties were resolved through discussion with a third reviewer.

## Results

### Study Characteristics

**Figure 1:**
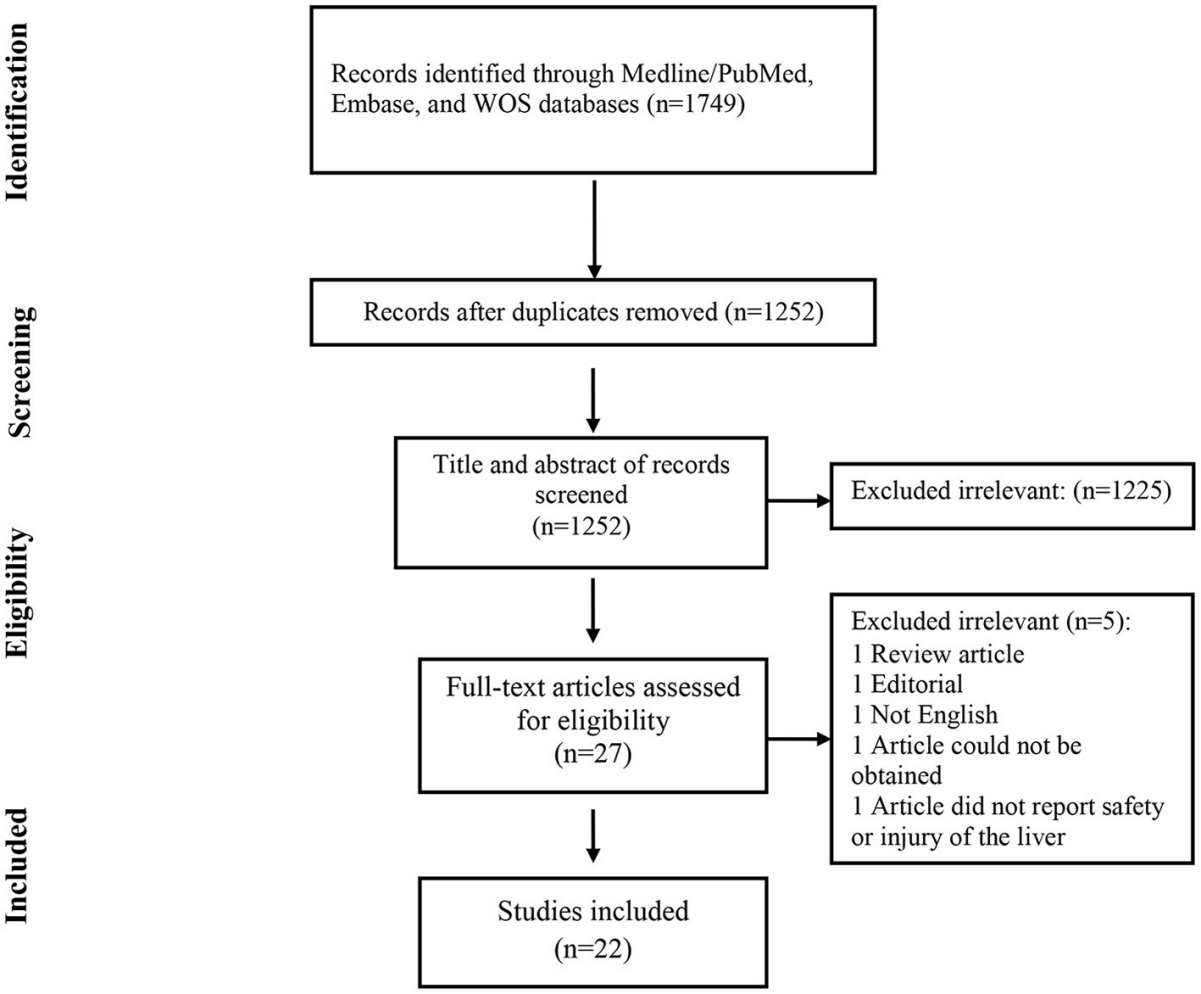
Flow chart of study selection for inclusion in the systematic review.

The selection process of articles is shown in Figure 1. Finally, we included 22 related articles. Among included studies, there was five case report, five case series, four randomizes control trial (RCT), seven cohort studies, and one cross-sectional study. In addition, six studies were from China, five studies from Italy, four studies from the USA, one study from Korea, one study from Brazil, one study from Ireland, one study from the Netherlands, one study from Montenegro, one study from India, and one study from Japan. The majority of included studies (11 studies) evaluated the safety of remdesivir for liver function, six studies reported safety of lopinavir/ritonavir, three studies assessed the safety of tocilizumab, three studies reported the safety of hydroxychloroquine. In addition, two studies showed the safety of favipiravir for liver function (Table 1).

**Table 1:** Characteristics of included studies.

**First Author**	**Country**	**Date of publication**	**Type of study**	**Sample size**	**Male: Female**	**Mean age**	**Nationality**	**MEDICATIONS**	**Inclusion criteria**	**Exclusion criteria**	**Severity of disease (mild, mod, severe)**	**Complications**	**COVID-19 symptoms**	**Discharge**
Fan et al. (17)	China	10-Apr-20	retrospective cohort study	148	73 M, 75 F	50	Chinese	lopinavir/ ritonavir	Clinical criteria of discharge and diagnosis were according to the standards for “Diagnosis and Treatment Scheme of New Coronavirus Infected Pneumonia” (trial version 6), history of exposure and most had clinical manifestations including fever or respiratory symptoms	–	severe (10)	–	fever in 127, cough in 67, Diarrhea in 6, Nausea and vomiting in 3, expectoration in 38, asymptomatic in 5, other liver disease in 9	92
Grein et al. (20)	USA	10-Apr-20	cohort, compassionate-use	53	40 M, 13 F	64	United States, Japan, Italy, Austria, France, Germany, Netherlands, Spain, Canada	remdesivir	COVID-19 infection confirmed by RT-PCR, needing O2 support or O2 Sat ≤ 94% while breathing ambient air, creatinine clearance > 30 mL/min, serum AST and ALT levels <5x ULN, patient consent to not use other investigational drugs(agents) for Covid-19.	missing post baseline information, an incorrect remdesivir start date	Severe	DM in 9, HTN in 13, Asthma in 6, Any coexisting condition in 36, Hyperlipidemia in 6	–	13
Cai et al. (21)	China	13-Apr-20	cross-sectional study	417	198 M, 219 F	49	–	Antibiotics, NSAIDs, Ribavirin, Oseltamivir, Herbal medications, Interferon, lopinavir/ritonavir	≥1 abnormal result of liver test from admission until end of February 2020	patients with hypertension at admission and found that the prevalence of abnormal liver function tests remained similar	severe (91), mild (326)	DM in 21, HTN in 51, Prior hepatic dysfunction in 19	fever in 248, cough in 131	–
Sun et al. (22)	China	20-Apr-2020	Retrospective cohort study	217	106 M, 111 F	45.7	Chinese	lopinavir/ritonavir, umifenovir	Confirmed SARS-CoV-2 patients	–	Severe (50)	In 62 patients (HTN, DM, HIV, CVD, CKD, COPD)	–	–
Wang et al. (23)	China	29-Apr-20	randomized controlled trial	158	89 M, 69 F	66	–	remdesivir	males and non-pregnant females with COVID-19 aged ≥ 18 years with positive RT-PCR test for SARS-CoV-2, confirmed pneumonia by chest imaging, O2 Sat ≤ 94% on room air or a ratio of arterial O2 partial pressure(PaO2) to fractional inspired O2 ≤ 300 mmHg, within 12 days of start of symptoms.	pregnancy or breast feeding; hepatic cirrhosis; ALT or AST > 5x ULN; known severe renal impairment (estimated GFR <30 mL/min per 1·73 m^2^) or receipt of continuous renal replacement therapy, haemodialysis, or peritoneal dialysis; possibility of transfer to a non-study hospital within 72 h; enrolment into an investigational treatment study for COVID-19 within 30 days before screening	severe	DM in 40, HTN in 72, coronary heart disease in 15	fever in 56	Day 28 clinical improvement in 103 of remdesivir group and in 45 of placebo group - Clinical improvement rates at days 14 and day 28 were also not significantly different between the groups, but numerically higher in the remdesivir group than the placebo group
Antinori et al. (24)	Italy	11-May-20	prospective (compassionate), open-label study	35	26 M, 9 F	63	Italian	remdesivir	males or non-pregnant females aged >18 years, with SARS-CoV-2 infection confirmed by RT-PCR of a respiratory tract sample and pneumonia confirmed by a chest X-ray or CT scan, mechanically ventilated or SaO2 level of <94% in room air or a National Early Warning Score (NEWS) 2 of ≥4	ALT or AST level >5x ULN, creatinine clearance <30 mL/min	–	DM in 3, HTN in 12, obesity in 3, cancer in 1	–	At day 28 from starting remdesivir, 14 patients were discharged from IDW, from ICU 6 discharged, 1 was improved but still hospitalized
Mangoni et al. (25)	Italy	16-May-20	case Report	4	4 M	52	Italian	remdesivir	–	–	severe	–	severe pneumonia and respiratory distress	3
Muhović et al. (26)	Montenegro	17-May-20	case report	1	1 M	52	Montenegrin	Tocilizomab	–	–	severe	–	fever, cough	–
Goldman et al. (27)	US	27-May-20	multicenter, randomized, open-label, phase 3 trial	397	253 M, 144 F	61.5	United States, Italy, Spain, Germany, Hong Kong, Singapore, South Korea, Taiwan	remdesivir	confirmed SARS-CoV-2 infection, age ≥ 12 years, O2 Sat ≤ 94% while breathing ambient air or O2 support, radiologic evidence of pneumonia	mechanical ventilation or extracorporeal membrane oxygenation (ECMO) at screening, ALT or AST > 5x ULN, creatinine clearance <50 mL/min, receiving simultaneous treatment (within 24 h before the start of trial treatment) with other agents with supposed activity against Covid-19	severe	DM in 90, HTN in 198, asthma in 49, hyperlipidemia in 89	–	84
Falcão et al. (28)	Brazil	1-Jun-20	case report	1	1 F	29	Brazilian	HCQ	–	–	severe	–	dry cough, severe dyspnea, weakness, one episode of hemoptysis	–
Jiang et al. (29)	China	23-Jun-20	Multicenter retrospective, observational study	131	70 M, 61 F	51.21 ± 6.1	Chinese	lopinavir/ritonavir	critically ill and non-critically ill	pregnancy, <18 years old, liver function abnormality before treatment	non-severe = mild or moderate (30), severe (22) critically ill (27)	Cardiovascular and cerebrovascular diseases in 37, endocrine system disease in 22, Digestive system disease in 5, Neurological disorders in 4, Immune system in 2	–	–
Guaraldi et al. (31)	Italy	24-Jun-2020	retrospective, observational cohort study	179	127 M, 52 F	64	–	tocilizumab	≥18 years with PCR confirmed COVID-19 on nasopharyngeal swab, eligible for tocilizumab treatment, if presented SaO2 <93% and a PaO2/FiO2 ratio <300 mm Hg in room air or a decline > 30% in them PaO2/FiO2 ratio in the last 24 h during hospitalization.	Exclusion criteria for tocilizumab use: concurrent infection other than COVID-19; a PaO2/FiO2 ratio > 300 mm Hg; chronic or current use of glucocorticoid; history of severe allergic reactions to monoclonal antibodies; <500 per μL neutrophils or <50 × 10^9^ platelets; active diverticulitis, inflammatory bowel disease, or another symptomatic digestive tract condition that might incline patients to perforation of bowel; severe liver, renal, or hematological function damage.	–	cancer in 2, renal insufficiency in 2	–	–
Leegwater et al. (32)	The Netherlands	28-Jun-20	case report	1	1 M	64	Dutch (the Netherlands)	remdesivir	–	–	severe	HTN, hypercholesterolemia	Fever, cough, headache, progressive dyspnea	1
Dubert et al. (33)	France	30-Jun-20	case series	5	5 M	59.2	Chinese, French	remdesivir	patients admitted to the Bichat-Claude Bernard University Hospital, Paris, France, between January 24 and March 1, 2020, diagnosed with COVID-19 and treated with remdesivir (GileadSciences), criteria for compassionate-use remdesivir defined by the French national regulatory authorities and French Ministry of Health: signs of severe illness at diagnosis or subsequent clinical worsening (respiratory symptoms or general signs)	–	ICU	Obesity in 1, malignancy in 1, pulmonary disease in 1, chronic kidney injury in 1	fever in 5, cough in 4, GI symptoms in 1	3
Kelly et al. (34)	Ireland	8-Jul-20	retrospective study	82	55 M, 27 F	64.8	–	HCQ+ azithromycin	–	–	–	–	–	–
Lee et al. (35)	Korea	23-Jul-20	retrospective case series	10	5 M, 5 F	52	Korean (ME: Korea, Philippines, UK)	remdesivir	confirmed diagnosis of SARS-CoV-2-related pneumonia, aged ≥18 years (or 12–18 years if weighed ≥40 kg) and O2 Sat of ≤ 94% in room air	evidence of multi-organ failure, mechanical ventilation > 5 days, serum AST or ALT >5x ULN, creatinine clearance <50 mL/min (Cockcroft-Gault formula if aged ≥18 years and Schwartz formula in aged <18 years),	3 in ICU	HTN in 3, FLD in 1, other comorbidities in 2	fever in 8, cough in 5, asymptomatic in 1, other symptoms in 10	3
										pregnancy or breastfeeding, known hypersensitivity to RDV or its metabolites, participation in another clinical trial			
Zampino et al. (36)	Italy	28-Jul-20	case series	5	5 M	51.2	Italian	remdesivir	invasive mechanical ventilation, ALT <5x ULN, creatinine clearance >30 mL/ min	Multi-organ failure, a need for vasopressor	–	HTN in 1, asthma in 1	–	Final outcome positive in 4/5 patients (maybe 4 discharged)
Hundt et al. (37)	USA	29-July-2020	Retrospective observational cohort	1,827	969 M, 858 F	64.6	–	lopinavir/ritonavir (*n* = 136), hydroxychloroquine (*n* = 1,469), remdesivir (*n* = 46), and tocilizumab (*n* = 772)	patients who tested positive for SARS-CoV-2 by PCR of nasopharyngeal swab	–	Non-severe (*n* = 1,175) Severe (*n* = 652)	DM in 712, Obesity in 748	–	–
Carothers et al. (38)	US	2-Oct-20	case series	2	2 F	74	–	remdesivir	–	–	–	DM in 2, HTN in 2, CAD in 1, hyperlipidemia in 2	fever in 1, oxygen saturation in the 70s and 82s, systolic blood pressure > 200 mm Hg in 1, chills in 2, fatigue in 2, body aches in 1, low back pain in 1, shortness of breath in 1, difficulty with urination in 1	1
Serviddio et al. (39)	Italy	7-Oct-20	case series	7	7 M	59	–	HCQ+ azithromycin+ lopinavir/ritonavir	–	–	mild (6), severe (2)	–	fever in 7	7
Aiswarya et al. (40)	India	18-Dec-2020	Observational prospective study	48	38 M, 10 F	50	–	remdesivir	patients with CKD needing hemodialysis, who had positive test for SARS-CoV-2 infection from nasopharyngeal swab by RT-PCR with moderate or severe infection, and who received ≥1 dose of remdesivir	Patients with milddisease and underlying chronic hepatic disease	Moderate (21), severe (27)	DM in 20, HTN in 41, all patients (*n*=48), CKD in all patients.	–	38
Yamazaki et al. (41)	Japan	28-Dec-2020	Case report	1	1 M	73	Japanese	favipiravir	–	–	severe	Alcoholic hepatitis, HTN, hyperlipidemia, gastric ulcer, BPH, anemia	–	–

*HTN, hypertension; CKD, chronic kidney disease; BPH, benign prostatic hyperplasia; FLD, fatty liver disease; DM, diabetes mellitus; CAD, coronary artery disease; CVD, cardiovascular disease; COPD, chronic obstructive pulmonary disease*.

### Drug-Induced Liver Injury

#### -Remdesivir

The first study reporting the safety of remdesivir for COVID-19 patients, conducted by Grein et al., investigated the effect of 5 to 10-days courses of remdesivir on the changes in the category of oxygen-support status in a small cohort of 53 patients. The most common adverse event in this study was increased hepatic enzymes by an incidence of 23%. Moreover, one of the four patients who discontinued the treatment was due to the elevated liver aminotransferase (20). A similar pattern was replicated in the study on 402 patients, evaluating the optimum time-course for intravenous remdesivir, conducted by Goldman et al. In that grade, 1-2 ALT and AST elevation (7 and 6% respectively) was reported as the most common liver adverse effects (27). Furthermore, in the placebo-controlled double-blinded clinical trial on a total sample of 255 patients, conducted by Wang et al., grade 1–2 Increased AST was detected as an adverse liver effect (12% in the placebo group, 7% in remdesivir group; or 12:7%) and grade 1–2 increased ALT led to drug discontinuation (1%). However, the most common liver adverse effects reported by the same study were grade 1–2 hypoalbuminemia (15:13%) and grade 1–2 increased bilirubin (9:10%) respectively, latter of which also caused drug discontinuation (1%) (23). In an exciting perspective open-labeled study, remdesivir induced adverse effects were compared between patients in an intensive care unit (ICU) and infectious disease wards (IDW). While aminotransferase elevation was almost equal among the two groups (ICU = 44.4%; IDW = 41.2%), bilirubin elevation was more probable in ICU rather than IDW patients, suggesting that the differences in the incidence of different adverse effects among further studies may be due to the different severity states of COVID-19 in the patients (24). In addition to this line of studies, there are also some case reports. In a recent one, an acute increase in ALT was reported after 2 days of remdesivir initiation and was corrected immediately following the stop of remdesivir (32). In two other case reports, hepatic enzyme elevation was detected in patients receiving remdesivir with or without HCQ, who were previously treated with lopinavir/ritonavir (35, 36). In another case report, Carothers et al. have suggested that the use of acetylcysteine can be beneficial in the management of acute liver failure (ALF) induced by remdesivir (38).

#### -Lopinavir/Ritonavir

A significant number of studies have reported the association of lopinavir/ritonavir to use in COVID-19 patients with adverse liver effects. In a study by Sun et al. on a sample of 217 patients, 63% of total adverse drug reactions (ADRs) were associated with the use of lopinavir/ritonavir, whereas the use of other drugs including umifenovir, chloroquine, and antibacterial drugs together accounted for the additional 47% of ADRs. Liver ADRs were the second common ADRs by a prevalence of 18%. However, the percentage of liver ADRs due to lopinavir/ritonavir was not reported by the same study (22). Later, Fan et al. reported that among the 148 patients, 45 patients had normal base-line liver functions of which, 48% developed an abnormality in the liver after admission to the hospital. They highlighted that among the patients with abnormal liver functions, a higher proportion had used lopinavir/ritonavir (57.8%) compared to the patients with normal liver function tests (31.3%) (17).

Furthermore, Cia et al. reported that liver dysfunction was significantly higher in lopinavir/ritonavir treated group in a study with 417 COVID-19 patients. A 4-fold magnitude increased liver function odds, and the most common increase in test results was observed in gamma-glutamyl transferase and total bilirubin. Yet, due to the lack of evidence supporting the role of drugs in observed liver injury, the definition of DILI by clinical guidelines from the “European Association for the Study of the Liver” was not applicable for this study (21). In another line of studies, Jiang et al. observed that adding each concomitant medication is followed by a 12.1% increase in odds of liver function (29). In addition, concomitant use of lopinavir/ritonavir and arbidol in non-critically ill COVID-19 patients increased the odds of liver functions more than expected, to 3.58 times greater who didn't receive the medications mentioned earlier. To find out the mechanism of this abnormal increase, metabolic interactions between the two medications were explored using human liver microsomes. In the following line of evidence, a case series of seven patients who showed significant abnormal liver tests in addition to worsening of the respiratory system function 5–7 days following the treatment with lopinavir/ritonavir, hydroxychloroquine, and azithromycin, use of tocilizumab was seen to relieve both lung and liver functions within 3 weeks (39).

#### -Tocilizumab

A retrospective study reported no adverse liver effects on 1,351 patients treated with tocilizumab conducted by Guaraldi et al. (31). However, there is a case report DILI following the use of tocilizumab, which was suggested to possibly be a result of previous use of lopinavir/ritonavir (26). Another study conducted by Hundt et al. reported a significant correlation between the use of lopinavir/ritonavir, hydroxychloroquine, remdesivir, and tocilizumab developing a liver injury. Furthermore, the strongest correlation was related to the use of tocilizumab (37).

#### -Hydroxychloroquine (+/-Azithromycin)

A retrospective analysis on a sample of 134 patients reported that the liver function tests were not significantly different between patients treated with hydroxychloroquine/azithromycin compared to the patients who didn't receive targeted therapies (34). However, Falcao et al. reported a severe COVID-19 case of hepatotoxicity (28) related to hydroxychloroquine. The patient showed a 10-fold increase in levels of transaminases in serum, which rapidly decreased after being withdrawn from hydroxychloroquine.

#### -Favipiravir

We found one study reporting the effect of favipiravir use on liver function. This case report described a patient who developed cholestatic liver injury caused by favipiravir. However, based on the author's view, the administration of antibacterial treatment triggered the liver injury, and a high dose of favipiravir worsened the liver function (41).

Table 2 provides a brief overview of the effects of the mentioned medications on the liver function tests.

**Table 2:** Effect of drugs on liver function tests.

**First author**	**Sample size**	**Medication**	**Abnormal liver tests**	**Proposed risk factor**
Fan et al. (17)	148	lopinavir/ ritonavir	ALT ↑ in 27, AST ↑ in 32, GGT ↑ in 26 ALP ↑ in 6, total bilirubin ↑ in 9, bilirubin ↑ in 18	Liver injury is attributed to lopinavir/ritonavir
Grein et al. (20)	53	remdesivir	Liver enzymes ↑ in 12	Liver injury is attributed to remdesivir.
Cai et al. (21)	417	Antibiotics, NSAIDs Ribavirin, Oseltamivir Herbal medications Interferon lopinavir/ritonavir	ALT ↑ in 167, AST ↑ in 137, Bilirubin↑ in 196 ALP ↑ in 71, GGT ↑ in 143	Liver injury is attributed to lopinavir/liponavir. Liver injury is not attributed to Antibiotics, NSAIDs Ribavirin, Oseltamivir Herbal medications, and Interferon.
Sun et al. (22)	217	lopinavir/ ritonavir, umifenovir	ALT ↑ in 30, Liver and biliary system disorders	Liver injury is attributed to lopinavir/ritonavir
Wang et al. (23)	158	remdesivir	ALT ↑ in 2, AST ↑ in 7, Bilirubin ↑ in 16 Albumin ↓ in 20, WBC ↑ in 11 Thrombocyte ↓ in 20, neutrophil ↑ in 10	Liver injury is attributed to remdesivir.
Antinori et al. (24)	35	remdesivir	Hypertransaminasemia ↑ 15, total bilirubin ↑ 7	Liver injury is attributed to remdesivir.
Mangoni et al. (25)	4	remdesivir	ALT & AST ↑ 3	Liver injury is attributed to remdesivir.
Muhovic et al. (26)	1	tocilizumab	ALT & AST ↑, Bilirubin normal, ALK normal	Liver injury is attributed to tocilizumab and previous use of lopinavir/ritonavir
Goldman et al. (27)	397	remdesivir	ALT ↑ in 26, AST ↑ in **23**/25, Bilirubin ↑ in 5, Creatinine clearance ↓ in 54, aminotransferase ↑ in 5	Liver injury is attributed to remdesivir.
Falcao et al. (28)	1	HCQ	ALT ↑ to 357 U/L, AST ↑ to 469 U/L, Bili normal, ALK normal, CRP↑ to 270 mg/L, GGT normal.	Liver injury is attributed to HCQ.
Jiang et al. (29)	131	lopinavir/ritovir	ALT ↑ in 45, AST ↑ in 41, Bilirubin ↑ in 43	Liver injury is attributed to lopinavir/ritovir.
Guaraldi et al. (31)	179	tocilizumab	–	Liver injury is not attributed to tocilizumab.
Leegwater et al. (32)	1	remdesivir	ALT & AST ↑	Liver injury is attributed to remdesivir.
Dubert et al. (33)	5	remdesivir	ALT ↑ in 2	Liver injury is attributed to remdesivir.
Kelly et al. (34)	82	HCQ+ azithromycin	LFT ↑	Liver injury is not attributed to HCQ+ azithromycin
Lee et al. (35)	10	remdesivir	ALT ↑ in 5, AST ↑ in 5, CRP ↓ in 10, LDH ↓ in 10	Liver injury is attributed to remdesivir.
Zampino et al. (36)	5	remdesivir	ALT ↑ in 4, AST ↑ in 4	Liver injury is attributed to remdesivir.
Hundt et al. (37)	1,827	lopinavir/ritonavir, hydroxychloroquine, remdesivir, and tocilizumab	AST ↑, ALT ↑, ALP ↑, total bilirubin ↑	Liver injury is attributed to lopinavir/ritonavir, hydroxychloroquine, remdesivir, and tocilizumab, with the strongest correlation with tocilizumab.
Carothers et al. (38)	2	remdesivir	ALT ↑ in 2, AST ↑ in 2, Bilirubin ↑ in 2, ALK ↑ in 1, amonia and INR ↑ in 2	Liver injury is attributed to remdesivir.
Serviddio et al. (39)	7	HCQ+ azithromycin+ lopinavir/ritonavir	ALT & AST & GGT ↑ in all (all patients experienced elevation after treatment with hqc, azithromycin and lopinavir/ritonavir, but after tocilizumib decreased)	Liver injury is attributed to HCQ+ azithromycin+ lopinavir/ritonavir
Aiswarya et al. (40)	48	remdesivir	serum CRP ↓, serum LDH, serum transaminases and ferritin no significant change,	Liver injury is not attributed to remdesivir.
Yamazaki et al. (41)	1	favipiravir	AST ↑, ALT ↑, total bilirubin ↑, GTP ↑, ALP ↑, LDH ↑	Liver injury is attributed to favipiravir

*ALT, alanine aminotransferase; AST, aspartate aminotransferase; ALP/ALK, alkaline phosphatase; ALK, alkaline; GGT, gamma-glutamyl transferase; GTP, gama-glutamyl transpeptidase; LDH, lactate dehydrogenase; CRP, C-reactive protein*.

## Discussion

Among included studies, some of theme revealed a direct role of drugs, while others couldn't certainly confirm that the liver injury was due to SARS-CoV-2 itself or administration of medications. However, a major number of studies reported that liver injury could be attributable to drug administration. Among included studies, one study reported that liver-related adverse effects were not significantly different between patients who used hydroxychloroquine (HCQ) and azithromycin and the control group (34). Another study, however, reported that DILI in COVID-19 is mainly attributed to the type of the drug. For instance, a study conducted in China by Cai et al. reported that drugs such as antibiotics, NSAIDs, ribavirin, herbal medication, and interferon did not significantly lead to a higher risk of liver injury. In contrast, drugs including lopinavir/ritonavir were associated with 4 × higher odds of liver injury (21). Of course it is noteworthy to imply that the efficacy of lopinavir/ritonavir in COVID-19 patients is still under question and should be evaluated in further studies (42, 43).

Furthermore, Chinese herbal medication and antibiotics are frequently related to DILI in China, but Chinese herbal medicine was not associated with liver injury (44). Therefore, it can be concluded that there is no consensus on what drugs could lead to DILI, particularly in the COVID-19 context. According to included studies, all papers that evaluated the adverse effects of remdesivir on the liver reported that remdesivir could lead to liver injury except for one recent article which demonstrated that RDV treatment was not associated with transaminase elevation (40). The most controversial reports of DILI were about the effect of tocilizumab on the liver. One study reported that tocilizumab improved liver adverse effects caused by the administration of lopinavir/ritonavir (39). Inconsistently, a study conducted by Muhovic et al. reported the first case of DILI caused by tocilizumab. According to this study, previous use of antiviral drugs such as lopinavir/ritonavir could increase the hepatotoxic effects of tocilizumab (26). Moreover, a retrospective cohort study conducted by Guaraldi et al. has demonstrated that tocilizumab does not increase transaminases in COVID-19 patients (31). Two meta- analysis studies, although incoherent about the efficacy of tocilizumab, have concluded that tocilizumab is not associated with liver injury in COVID-19 patients (45, 46). Also it has been reported that a combination of tocilizumab with other hepatotoxic agents could lead to severe liver injury (26). Developing DILI in patients is associated with various factors. For instance, Falcao et al. reported that a high dose of recommended HCQ could increase the risk of hepatotoxicity in COVID-19 patients. Moreover, certain medical conditions such as porphyria cutanea tarda, viral hepatitis, and rheumatologic diseases could enhance the risk of liver injury development (28). Further, there is a drug-drug interaction with chloroquine and its derivatives with anti-rejection immunosuppressant (47). Based on a meta-analysis, the incidence of DILI in a population of 208 patients treated with remdesivir was 15.2%, while the incidence of DILI among 775 patients treated with lopinavir/ritonavir was 37.2% (5). According to a meta-analysis conducted by Yadav et al., severe cases and patients with liver injury are at higher risk of mortality therefore, during treatment, they should be given special and careful attention (48).

### Liver Involvement in COVID-19 Infection

SARS-COV-2 enters the host cells through the angiotensin 2 conversion enzyme (ACE2) receptor. This receptor is expressed in various tissues, including lungs, the heart, and the liver. ACE2 receptor in the liver is highly expressed in cholangiocytes (60%) and hepatocytes (3%), indicating that the liver could be a potential target for SARS-CoV-2 invasion (49).

Liver involvement during COVID-19 infection is associated with various factors. Several mechanisms have been postulated about liver involvement in COVID-19 infection that can be listed as: (1) direct invasion of the virus to liver cells through ACE2 receptor, (2) uncontrolled inflammatory responses that lead to fibrosis and liver dysfunction, (3) liver dysfunction caused by administration of anti-COVID-19 drugs, (4) hypoxia and cardiac failure in severe COVID-19 patients could contribute to the development of liver injury (50).

### Drug-Induced Liver Injury

Drug-induced liver injury (DILI) is liver lesion/dysfunction caused by medication. The incidence of DILI is low; however, it could lead to acute liver failure and urgent liver transplantation. In patients with acute liver failure, DILI is a differential diagnosis (49). To better diagnose DILI in suspected patients, the potential hepatotoxic effect of drugs and various influential factors including race, age, and sex should be considered (51). In patients with COVID-19, the cause of liver dysfunction should be determined. Furthermore, taking appropriate measures such as ALT, AST, total bilirubin, direct bilirubin, albumin, and INR monitoring could significantly reduce morbidity and mortality. Moreover, patients with DILI should be given anti-inflammatory liver protection medication, and special attention should be considered to alter the dosage or discontinue the suspected drugs (49). In patients with severe COVID-19 infection and patients with pre-existing liver diseases, too many drugs (more than 2) with the potential of hepatotoxic effect should not be given. Drugs in patients with ongoing anti-HBV and anti-HCV should not be discontinued; but instead, they should be carefully monitored (49).

Definition of acute liver injury is based on the ULN of serum concentration of ALT, AST, and total bilirubin. It is as follows: 1 increase level of ALT ≥5-times ULN, or increase level of ALP ≥2-times ULN (in the absence of bone pathology), or simultaneous increase of ALT ≥3-times ULN and total bilirubin concentration >2-times ULN (52).

It has been reported that patients who developed favipiravir (FRP)-induced liver damage had higher FRP serum levels than patients who did not (53). Practitioners should notice a large variation in FRP concentration between patients; therefore, monitoring FPR concentration in patients' blood and personalized FPR dosing could be helpful. Administration of FRP could cause the enhanced level of ALT, AST, ALP, and total bilirubin. In the context of COVID-19 infection, ALT elevation with the use of FPR occurs in <10% of patients (54). It should be considered that in patients with severe COVID-19 infection reduced dosage of FPR should be administered (55). A review on the safety and efficacy of FRP in COVID-19 patients revealed that there is not a significant difference in LFT changes in the FRP group compared to the comparison group (56). Consistently, a recent meta-analysis study reported FRP leads to non-significantly lower odds for adverse effects compared to placebo (57).

Studies have reported that administration of RDV is associated with AST and ALT elevation (36). Consistently, according to our included studies, the most important changes of liver enzymes were altered levels of ALT and ST. However, in most cases, elevated levels of AST and ALT do not progress to severe liver injury (58). Based on reports, liver injury caused by RDV occurred in two patients manifested with increased transaminases, coagulopathy, and hepatic encephalopathy that occurred between days 3 and 10 of RDV administration. Practitioners used N-acetyl cysteine and discontinued RDV to stop the progression of acute liver failure (38). It should be considered that in the following conditions RDV should be stopped; ALT >5-times ULN or ALP >2-times ULN, and total bilirubin >2-times ULN or presence of coagulopathy or clinical decompensation (54). To reduce RDV-induced liver adverse effects, liver function tests should be performed and analyzed before drug initiation. Moreover, physicians should monitor the liver function tests during treatment with RDV (59). Carothers et al. have suggested that the use of acetylcysteine can be beneficial in the management of acute liver failure (ALF) induced by remdesivir (38). Acetylcysteine is an antidote to acetaminophen, the leading cause of ALF, which is possibly useful also for ALF caused by drugs other than acetaminophen (60).

Azithromycin could cause idiosyncratic acute liver damage. azithromycin-induced liver injury manifested with cholestatic hepatitis occurring 1–3 weeks after treatment initiation. Moreover, hepatocellular injury associated with azithromycin has a short latency (52). azithromycin is also known to develop cutaneous reactions, including erythema multiform and Stevens-Johnson syndrome, that are often associated with a degree of liver involvement (61).

Moderate to severe elevation in serum concentration of aminotransferases (>5-times UNL) could be seen in 3–10% of patients who have used lopinavir. The extent of liver injury varies from hepatocellular injury to cholestatic injury or both (62). Elevation of liver enzymes following the use of ritonavir is rare and self-limited. Moreover, the administration of lopinavir/ritonavir could exacerbate liver dysfunction in patients with HBV and HCV infection (52).

We did not include ivermectin (IVN) and colchicine into our search strategy but as there is a tendency to investigate the possible usefulness of these two medications in COVID-19 treatment, we should point them. IVN, a well-known anti- parasite medication, is considered a safe drug, and reports on its hepatotoxic effects are rare. There are growing and controversial evidences about IVN efficacy in treatment of COVID-19 patients but it seems to be a safe medication in overall (63–66). Of course there is a case report of IVN caused DILI (elevated aminotransferase, acute hepatocellular necrosis, lobular infiltration of lymphocytes, and without fibrosis) 1 month after drug administration and the patient clinically improved after 3 months (67). Colchicine is also another well-known drug used as an anti-inflammatory agent in wide range of diseases that has also been reported to reduce the severity, hospitalization period, and the mortality of COVID-19 and prevention of cytokine storm (68–71). But excessive cautions should be take place in colchicine dosage as it is easily affected by many factors (72, 73). Currently there are not enough data to comment on the effects of colchicine on the liver function of COVID-19 patients and further studies are recommended to elucidate it.

Practitioners could better diagnose the DILI in suspected patients based on Roussel Uclaf Causality Assessment Method (RUCAM). RUCAM is a structured, standardized, and validated method for the assessment of DILI. However, a major number of related studies did not use RUCAM to evaluate the liver damage and assess the risk of DILI, causing confounding results in the diagnosis of DILI in COVID-19 patients. Therefore, RUCAM could provide an accurate quantitative casualty grading for suspected drugs and verify DILI in suspected patients (30).

### Mechanisms of Drug-Induced Liver Injury

The mechanisms underlying liver injury in COVID-19 patients are not yet fully understood; however, drug-induced liver injury has been significantly cited in the literature among various causes of liver damage in COVID-19.

Xu et al. have reported moderate micro vesicular steatosis in post-mortem liver tissue of a COVID-19 patient (74), a condition in which hepatocytes are filled with fat vesicles due to either viral- or drug-induced injury. Drug-induced steatosis is mainly caused by drug interference with β-oxidation of fatty acids, mitochondrial respiration, or both (75), resulting in the accumulation of non-esterified fatty acids which are subsequently converted into triglycerides (76).

Several mechanisms are known to sensitize hepatocytes to SARS-CoV-2 infection or therapeutic chemicals. Underlying diseases including diabetes type 1 or 2 and hypertension, would enhance direct SARS-CoV-2 hepatotoxicity due to upregulation of ACE2 following the use of angiotensin receptor blockers or ACE inhibitor drugs (77, 78). Non-alcoholic fatty liver disease (NAFLD), which can be resulted from diabetes itself, sensitizes hepatocytes to therapeutic chemicals, specifically antipyretic drugs containing acetaminophen (79, 80).

The other mechanism thought to affect hepatocytes is downregulation of cytochromes p450 or CYPs family; enzymes involved in oxidative biotransformation of many drugs including the ones used in COVID-19 management. This downregulation is thought to be caused by the elevation of cytokines and interleukins especially IL-6, which is a major inflammatory mediator exerting repressive effects on several CYPs, as a result of cytokine storm syndrome (CSS) in COVID-19. Possible CYPs downregulation can affect the metabolism of several COVID-19 drugs, specifically remdesivir, the metabolism of which is extensively relied on CYPs. It's also thought that consuming multiple drugs would add up metabolic complexity to this situation (81).

As an example, arbidol and lopinavir are metabolized by Cytochrome P3A (CYP3A) which can be inhibited by ritonavir, hence using arbidol with lopinavir/ritonavir (LPV/RTV) at the same time may result in liver injury. It is concluded that the interaction between arbidol and lopinavir/ritonavir, elevates the serum concentration of arbidol and lopinavir and increases the risk of liver damage (29). Ribavirin causes hemolysis which in turn can aggravate or induce tissue hypoxia; this condition may lead to elevated levels of liver enzymes in the serum (82).

Almost all of the drugs prescribed for COVID-19, such as oseltamivir, lopinavir/ritonavir, ribavirin, chloroquine phosphate, and hydroxyl chloroquine sulfate are metabolized in the liver, hence liver damage and elevation of liver enzymes following the treatment is predictable (83). Pharmacological features of anti-COVID-19 drugs might elevate the risk of liver damage; lipophilicity, liability in the mitochondria, generation of reactive metabolites, the metabolism pathway in the liver, and the ability to inhibit hepatic transporters are some of the critical features that can lead to hepatotoxicity in susceptible hosts (84).

In a previous study by Griffin et al., 10-min exposure of rat hepatocytes to LPV and RTV, two protease inhibitor (PI) drugs, caused intracellular accumulation of Taurocholic Acid (TCA), suggesting that hepatotoxicity induced by PIs may be a result of their interference with the efflux of bile acids from hepatocytes (85). In addition, a series of studies have reported LPV as an important inhibitor of multidrug resistance–associated protein 2 (MRP2), an apical efflux transporter in hepatocytes, contributing to the excretion of bile acids (86, 87). In a more recent study evaluating the biliary excretion index (BEI) of 5(6)-carboxy-29, 79 dichlorofluorescein (CDF) through confocal imaging, the inhibitory effects of LPV was further supported (88). In another recent study, hepatotoxicity of PIs, LPV and, RTV, was reported to interfere with ER-Golgi trafficking via inhibition of Ras converting CAAX endopeptidase-1 (RCE1) and its potential substrates, leading to cellular stress responses and fatty liver disease (89). Another possible cause of hepatotoxicity due to LPV/RTV is insufficient P450 activity to metabolize large amounts of those drugs during the treatment period. Additionally, LPV/RTV can reactivate the infections caused by hepatitis B and C viruses and lead to deterioration of the liver disease (90).

Tocilizumab causes liver damage through not well-understood mechanisms but its ability to block IL-6, which is an important factor in the regeneration of the liver might be the underlying mechanism of mild-to-moderate liver enzyme elevation (90). IL-6 is also known to reduce viral entry to the host cells through downregulation of the Na(+)/taurocholate co-transporting polypeptide (NTCP), as well as viral replication in HBV infected patients (91, 92).

Consuming hydroxychloroquine in patients with porphyria cutanea tarda is associated with acute hepatotoxicity but the underlying mechanism is yet to be understood (90). Rismanbaf et al., postulated that synergy between inflammatory response to COVID-19 infection and adverse reaction to the reactive metabolite of HCQ could lead to liver injury (93).

The degree of lipophilicity, metabolization by CYP3A4 in the liver, inhibition of organic anion transporting polypeptide 1B1 (OATP1B1), p-glycoprotein (P-gp) and breast cancer resistance proteins (BCRP), all of which serve as transporters in the liver to protect it from xenobiotics, in addition to the activity of bile salt export pump (BSEP) which is involved in cholestasis process, are determining factors in estimating the hepatotoxicity of JAK inhibitors, including baricitinib, tofacitinib, upadacitinib, and ruxocitinib. In contrast to baricitinib which does not meet the criteria for hepatotoxicity in humans, tofacitinib and upadacitinib are known as more hepatotoxic agents especially in patients with underlying liver diseases or those who receive other potentially hepatotoxic drugs (84).

Furthermore, inflammatory response to the antivirals might be another probable cause of drug hepatotoxicity in COVID-19 patients (94).

## Conclusions

In conclusion, to the best of our knowledge, this is the first study that has assessed the drug-induced liver injury in COVID-19 infected patients. Liver injury in COVID-19 patients could be caused by the virus itself or the administration of some types of drug. Intensive liver function monitoring should be considered for patients, especially patients who are treated with drugs such as remdesivir, lopinavir/ritonavir, and tocilizumab.

## Limitations

There were limited studies that reported complete and detailed data about the safety and efficacy of drugs on liver function tests. Some of the studies did not certainly report that liver injury is due to drugs and they just raise the possibility of the drug's role in the development of DILI. Some of the articles did not report DIDI based on the RUCAM and other well-known validated methods of DILI assessment. Some of the articles reported liver-related adverse events and did not determine the degree of liver injury. More studies, particularly randomized clinical trials (RCTs) are required to better understand the risk of DILI following administration of these drugs.

## Data Availability Statement

The original contributions presented in the study are included in the article/supplementary material, further inquiries can be directed to the corresponding authors.

## Author Contributions

FS, ZS, NK, ME, and SN contributed in title screening, abstract screening, and full text screening. FS extracted full texts data. MN, MM, and YF conducted discussion part. MN and YF conducted second check of each steps. All authors contributed in writing the article.

## Conflict of Interest

The authors declare that the research was conducted in the absence of any commercial or financial relationships that could be construed as a potential conflict of interest.

## Publisher's Note

All claims expressed in this article are solely those of the authors and do not necessarily represent those of their affiliated organizations, or those of the publisher, the editors and the reviewers. Any product that may be evaluated in this article, or claim that may be made by its manufacturer, is not guaranteed or endorsed by the publisher.
